# A comprehensive analysis of vitamin a deficiency burden and trends: insights from the global burden of disease study 2021 and future predictions to 2050

**DOI:** 10.3389/fnut.2025.1673576

**Published:** 2025-11-18

**Authors:** Qianhong Hu, Junying Lyu, Jianying Li, Xiaoqing Lin, Sisi Li, Yiming Bu, Qianlei Zhao

**Affiliations:** 1 Department of Pediatrics, The Second Affiliated Hospital and Yuying Children's Hospital of Wenzhou Medical University, Wenzhou, Zhejiang, China; 2 Department of Pediatrics, The Second School of Medicine of Wenzhou Medical University, Wenzhou, Zhejiang, China

**Keywords:** nutritional deficiencies, vitamin A deficiency, global burden of disease, future predictions, public health

## Abstract

**Background:**

Vitamin A deficiency (VAD) continues to pose a significant public health challenge, particularly in low socio-demographic index (SDI) regions, contributing to increased infections, blindness, and child mortality. Its causes include poor diets, absorption issues, and infectious diseases.

**Objective:**

To comprehensively assess the global trends and burden of Vitamin A Deficiency from 1990 to 2021 and to forecast the global VAD-related burden from 2022 to 2050.

**Methods:**

This investigation employed data from the GBD 2021 database to analyze the global burden of nutritional deficiencies attributable to VAD. Disability-adjusted life-years (DALYs) and years lived with disability (YLDs) were systematically evaluated by gender, age groups, geographic regions, and countries over the 32-year study period (1990–2021). Temporal trends in age-standardized DALYs rate (ASDR) and age-standardized YLDs rate (ASYR) were quantified using estimated annual percentage change (EAPC) analysis. Projections of future burden were generated using exponential smoothing (ES) and autoregressive integrated moving average (ARIMA) models.

**Results:**

In 2021, the global burden of VAD was 1,104,931 DALYs, down from 1,970,337 in 1990. The ASDR dropped from 32.56 in 1990 to 15.73 in 2021, with an EAPC of −2.81%. Males had higher ASDR than females, both showing significant declines. The greatest burden was in the <5-year-old age group. An inverse SDI-ASDR associations was evident, with high-SDI regions achieving the fastest rate reductions. Projections to 2050 using ARIMA and ES models predicted a continued decrease in DALYs, with faster declines in males, although the ES model suggested a stabilization in DALYs for both genders.

**Conclusion:**

The GBD 2021 data underscored the persistent global challenge of addressing VAD. Solving this issue necessitates comprehensive strategies that combine targeted interventions, policy reforms, and collaborative efforts.

## 1 Introduction

Nutritional deficiencies (NDs) continue to pose a substantial global health burden, representing major contributors to morbidity, mortality and disability worldwide (1–3). Within the spectrum of NDs, Vitamin A deficiency (VAD) persists as a significant global health concern, disproportionately affecting populations in low- and middle-income countries (LMICs), where malnutrition is a persistent issue (4). As an essential micronutrient, vitamin A regulates various fundamental biological functions, including visual phototransduction, immune function, cellular differentiation, and reproduction (5, 6). Severe VAD significantly elevates mortality risk, particularly among children under 5 years old, and can lead to irreversible blindness due to xerophthalmia, increased susceptibility to infections, and poor maternal health outcomes (7–9). Despite extensive global efforts to combat VAD, it continues to impose a substantial burden on vulnerable populations, necessitating targeted interventions and continuous monitoring (10).

The etiology of VAD is multifactorial, primarily stemming from inadequate dietary intake, poor absorption due to gastrointestinal diseases, and increased metabolic demand during periods of rapid growth, infection, or pregnancy (11–13). In many LMICs, diets are heavily reliant on staple foods with low vitamin A content, contributing to widespread deficiency (14, 15). Additionally, infectious diseases such as diarrheal illnesses and measles exacerbate vitamin A depletion, further amplifying both morbidity and mortality risks (16–18). The interplay between malnutrition and infectious diseases highlights the need for integrated health policies that address both dietary deficiencies and disease prevention (19).

The Global Burden of Disease (GBD) Study has played a pivotal role in quantifying the worldwide burden of morbidity and mortality related to NDs. Previous analyses, including the GBD 2019 study, demonstrated a decline in both the global age-standardized incidence rate (ASIR) and age-standardized DALY rates (ASDR) for VAD worldwide, with estimated annual percentage changes (EAPC) of −3.11% (95% *CI*: −3.24 to −2.94%) and −2.18% (95% *CI*: −2.38 to −1.93%), respectively (1, 20). However, reductions were least pronounced in low sociodemographic index (SDI) regions, which continued to bear the highest burden of VAD globally. Epidemiological data from 2019 revealed Sub-Saharan Africa, and specifically the central region, exhibited the highest ASIR and ASDR in 2019, with Somalia and Niger ranking highest at the national level (10). Additionally, gender-disaggregated analysis revealed VAD remained disproportionately higher among males compared to females, with under-five children in low-SDI regions being the most affected demographic (21).

As the GBD database is updated to include 2021 data, it is imperative to reassess these trends, identify emerging disparities, and anticipate future challenges in the context of global demographic shifts, climate change, and economic instability (22). A nuanced understanding of these evolving patterns will be critical in refining public health strategies and ensuring more targeted and equitable interventions (23).

Utilizing the most recent Global Burden of Disease 2021 dataset, this study provides an updated reassessment of the global NDs burden and its predictive trends through 2050. Our objectives are threefold: (1) to quantify the DALYs of NDs attributable to VAD globally and stratified by demographic strata (age, gender, SDI region and nations) in 2021, identifying population and regions at greatest risk of VAD; (2) to assess VAD-related burden trends from 1990 to 2021; (3) to forecast the global VAD-related burden from 2022 to 2050. This analysis not only updates prior estimates but also explores the impact of recent global crises on nutritional outcomes, offering evidence to guide targeted policy interventions.

## 2 Methods

### 2.1 Data source

The GBD 2021 study (accessible via https://vizhub.healthdata.org/gbd-results/) systematically gathers and analyzes up-to-date global disease burden data encompassing 371 diseases and injuries, while simultaneously estimating the associations between 88 risk factors and health outcomes (24, 25). The disability-adjusted life-years (DALYs) in this study, including years lived with disability (YLDs) for VAD -related nutritional disorders, were sourced exclusively from the GBD 2021 database.

### 2.2 Descriptive analysis

In this study, we examined the distribution characteristics of the burden of NDs attributable to VAD globally and across different genders, age groups, regions and countries in 1990 and 2021. The formula for age-standardized rate (ASR) calculation is as follows:


$$\mathrm{ASR}=\frac{\sum_{i=1}^{A}a_{i}w_{i}}{\sum_{i=1}^{A}w_{i}}\times 100,000$$

Where i denotes the i th age group, $a_{i}$ represents age-specific rate, $w_{i}$ is the number of population (or weight) in the corresponding age groups of the selected reference standard population (26). Uncertainty intervals (UIs) were estimated based on the 2.5th and 97.5th percentiles derived from a 1,000-draw distribution for each metric (27). The SDI is a composite metric to evaluate a country or region’s overall socio-demographic development level based on fertility rates, educational attainment, and per capita income. It was used to classify regions and facilitate comparable analyses of disease burden. Countries and territories were classified into five groups according to their SDI scores as follows: low (SDI < 0.46), low-middle (SDI 0.46–0.60), middle (SDI 0.61–0.69), high-middle (SDI 0.70–0.81), and high (SDI > 0.81) (28). All statistical analyses were performed using R software (version 4.1.0), applying statistical significance defined as *p* < 0.05.

### 2.3 Trend analysis

The average trends in age-standardized DALYs rate (ASDR) and age-standardized YLDs rate (ASYR) during 1990 to 2021 were assessed using the estimated annual percentage change (EAPC). The formula for calculating EAPC is as follows:


y=α+βx+ε

EAPC=100×(exp(β)−1)

Where y represents ln(ASR) , x denotes the calendar year and β represents the slope obtained from the linear regression of the natural logarithm of the ASR on the year (29).

### 2.4 Forecasting analysis

The autoregressive integrated moving average (ARIMA) model is particularly effective in capturing trends and seasonal patterns in data, while the exponential smoothing (ES) model prioritizes recent observations, providing a comprehensive outlook on potential future developments (30). To ensure comprehensive and multi-perspective forecasting of the potential trends in the burden of VAD-attributable NDs from 2022 to 2050, we employed both ARIMA and ES methods. This dual approach was adopted as each method possesses distinct advantages in capturing different patterns in time series data, thereby providing complementary insights and enhancing the robustness of our projections. Statistical computing software R (Version 4.4.1) was used for all analyses and graphical representations.

## 3 Results

### 3.1 Global burden

**Figure 1:**
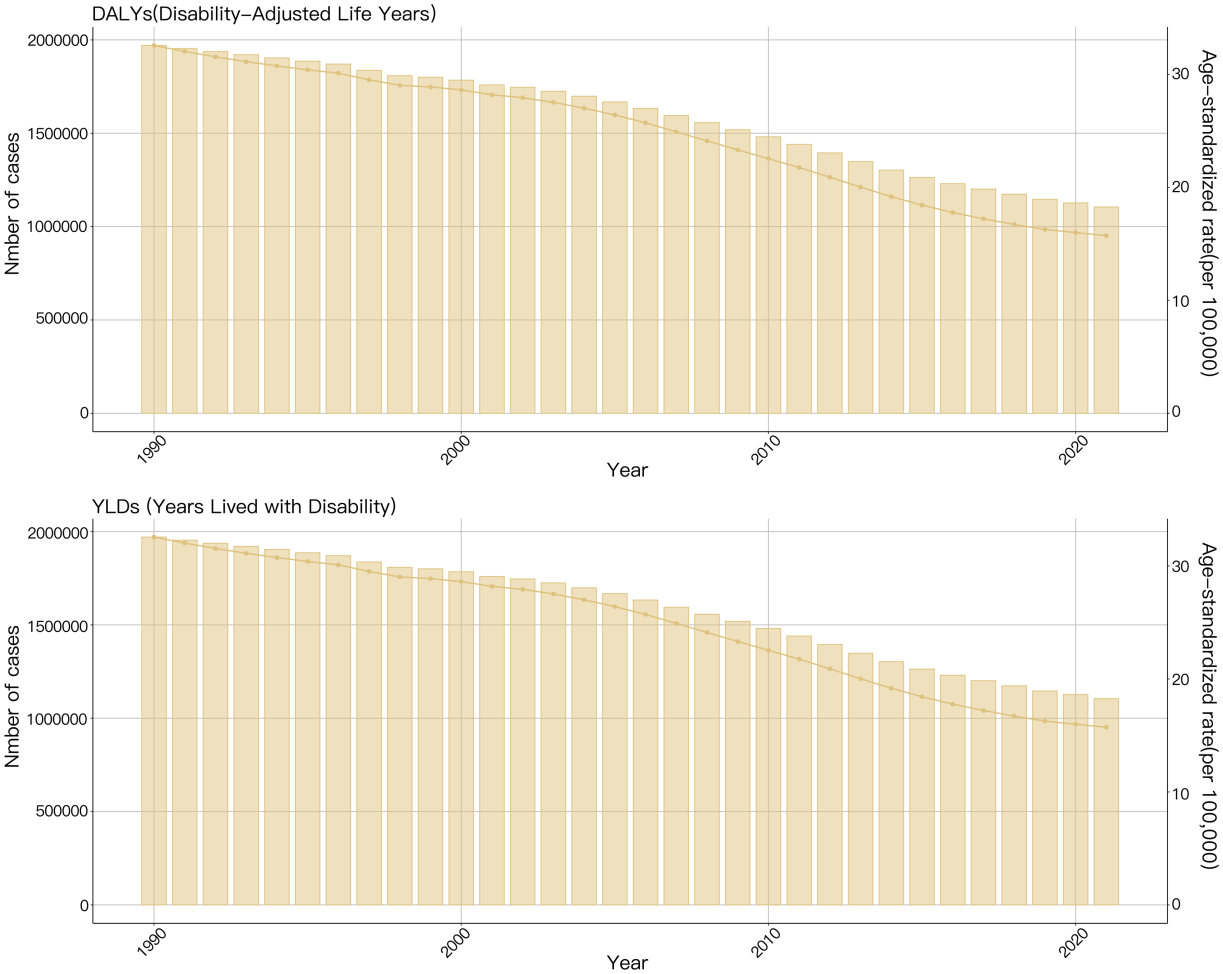
The global burden of nutritional deficiencies attributable to vitamin A deficiency from 1990 to 2021.

In 2021, there were 1,104,931 (95% UI: 711,387–1,561,756) DALYs caused by NDs attributable to VAD worldwide, representing a decrease from 1,970,337 (95% UI: 1,316,355–2,813,198) in 1990 (Figure 1). Between 1990 and 2021, the ASDR of VAD-attributable NDs decreased from 32.56 (95% UI: 21.77–46.45) in 1990 to 15.73 (95% UI: 10.09–22.28) in 2021, with a consistent downward trend (EAPC: −2.81, 95% *CI*: −2.96 to −2.66) (Figure 1). Detailed information on the disease burden of malnutrition is provided in Tables 1, 2.

**Table 1:** The DALYs and ASDR of nutritional deficiencies attributable to vitamin A deficiency in 1990 and 2021.

Characteristics	1990		2021		EAPC (95% *CI*)
	Number (95% UI)	ASDR (95% UI)	Number (95% UI)	ASDR (95% UI)	
Global	1,970,337 (1,316,355–2,813,198)	32.56 (21.77–46.45)	1,104,931 (711,387–1,561,756)	15.73 (10.09–22.28)	−2.81 (−2.96 to −2.66)
Sex					
Female	809,450 (538,870–1,138,427)	27.55 (18.35–38.74)	507,966 (328,327–731,843)	14.8 (9.59–21.42)	−2.11 (−2.29 to −1.92)
Male	1,160,887 (776,548–1,696,379)	37.29 (24.94–54.38)	596,965 (379,571–838,873)	16.6 (10.58–23.33)	−2.68 (−2.88 to −2.48)
Age					
<5 years	1,058,134 (717,792–1,504,884)	170.68 (115.78–242.75)	478,594 (308,327–691,737)	72.72 (46.85–105.1)	−2.99 (−3.25 to −2.72)
5–9 years	514,932 (332,272–756,468)	88.24 (56.94–129.64)	285,199 (173,150–427,960)	41.51 (25.2–62.29)	−2.36 (−2.57 to −2.15)
10–14 years	303,481 (197,224–444,029)	56.65 (36.82–82.89)	183,585 (121,444–266,349)	27.54 (18.22–39.95)	−2.18 (−2.32 to −2.04)
15–19 years	18,415 (10,562–27,687)	3.55 (2.03–5.33)	24,638 (14,300–36,510)	3.95 (2.29–5.85)	0.33 (0.28–0.39)
20–24 years	14,816 (8,418–22,748)	3.01 (1.71–4.62)	19,927 (11,545–30,262)	3.34 (1.93–5.07)	0.27 (0.21–0.32)
25–29 years	11,981 (7,059–18,029)	2.71 (1.59–4.07)	17,581 (10,170–26,912)	2.99 (1.73–4.57)	0.27 (0.21–0.32)
30–34 years	9,555 (5,741–14,387)	2.48 (1.49–3.73)	15,992 (9,461–23,819)	2.65 (1.57–3.94)	0.26 (0.21–0.3)
35–39 years	8,472 (5,205–12,762)	2.41 (1.48–3.62)	14,036 (8,508–21,407)	2.5 (1.52–3.82)	0.21 (0.18–0.25)
40–44 years	6,818 (4,061–10,556)	2.38 (1.42–3.68)	12,301 (7,375–19,211)	2.46 (1.47–3.84)	0.12 (0.07–0.16)
45–49 years	5,874 (3,580–9,075)	2.53 (1.54–3.91)	11,788 (6,906–18,435)	2.49 (1.46–3.89)	0.06 (−0.01 to 0.13)
50–54 years	5,019 (3,068–7,753)	2.36 (1.44–3.65)	10,903 (6,555–16,773)	2.45 (1.47–3.77)	0.15 (0.1–0.2)
55–59 years	4,261 (2,655–6,397)	2.3 (1.43–3.45)	9,426 (5,775–14,102)	2.38 (1.46–3.56)	0.15 (0.08–0.22)
60–64 years	3,310 (2,067–5,000)	2.06 (1.29–3.11)	7,271 (4,390–11,099)	2.27 (1.37–3.47)	0.23 (0.14–0.33)
65–69 years	2,332 (1,351–3,573)	1.89 (1.09–2.89)	5,764 (3,219–9,014)	2.09 (1.17–3.27)	0.46 (0.33–0.58)
70–74 years	1,510 (886–2,254)	1.78 (1.05–2.66)	3,884 (2,170–5,903)	1.89 (1.05–2.87)	0.55 (0.44–0.66)
75–79 years	853 (531–1,267)	1.39 (0.86–2.06)	2,259 (1,359–3,385)	1.71 (1.03–2.57)	0.66 (0.58–0.73)
80–84 years	405 (252–603)	1.15 (0.71–1.7)	1,174 (724–1,812)	1.34 (0.83–2.07)	0.62 (0.51–0.73)
85–89 years	136 (83–206)	0.9 (0.55–1.37)	465 (281–710)	1.02 (0.61–1.55)	0.33 (0.2–0.46)
90–94 years	29 (16–45)	0.67 (0.38–1.05)	120 (67–193)	0.67 (0.38–1.08)	−0.03 (−0.22 to 0.16)
95 + years	5 (2–9)	0.5 (0.25–0.85)	24 (11–42)	0.44 (0.21–0.78)	−0.16 (−0.36 to 0.04)
SDI region					
Low SDI	636,329 (433,125–916,268)	78.9 (53.42–113.22)	563,501 (366,834–798,764)	36.31 (23.71–51.36)	−2.66 (−2.85 to −2.47)
Low-middle SDI	856,153 (568,950–1,228,087)	52.67 (35.03–75.39)	355,962 (228,076–513,914)	18.09 (11.61–26.15)	−3.52 (−3.68 to −3.36)
Middle SDI	393,770 (258,654–555,974)	20 (13.18–28.22)	156,311 (101,981–224,243)	7.4 (4.83–10.66)	−3.24 (−3.32 to −3.17)
High-middle SDI	74,340 (49,352–106,572)	7.78 (5.17–11.16)	26,223 (16,923–37,814)	2.72 (1.69–3.95)	−3.62 (−3.75 to −3.5)
High SDI	8,565 (5,445–12,908)	1.32 (0.84–1.99)	2,247 (1,421–3,295)	0.3 (0.19–0.46)	−4.65 (−5 to −4.3)

**Table 2:** The YLDs and ASYR of nutritional deficiencies attributable to vitamin A deficiency in 1990 and 2021.

Characteristics	1990		2021		EAPC (95% *CI*)
	Number (95% UI)	ASYR (95% UI)	Number (95% UI)	ASYR (95% UI)	
Global	1,970,337 (1316355–2,813,198)	32.56 (21.77–46.45)	1,104,931 (711,387–1,561,756)	15.73 (10.09–22.28)	−2.81 (−2.96 to −2.66)
Sex					
Female	809,450 (538,870–1,138,427)	27.55 (18.35–38.74)	507,966 (328,327–731,843)	14.8 (9.59–21.42)	−2.11 (−2.29 to −1.92)
Male	1,160,887 (776,548–1,696,379)	37.29 (24.94–54.38)	596,965 (379,571–838,873)	16.6 (10.58–23.33)	−2.68 (−2.88 to −2.48)
Age					
<5 years	1,058,134 (717,792–1,504,884)	170.68 (115.78–242.75)	478,594 (308,327–691,737)	72.72 (46.85–105.1)	−2.99 (−3.25 to −2.72)
5–9 years	514,932 (332,272–756,468)	88.24 (56.94–129.64)	285,199 (173,150–427,960)	41.51 (25.2–62.29)	−2.36 (−2.57 to −2.15)
10–14 years	303,481 (197,224–444,029)	56.65 (36.82–82.89)	183,585 (121,444–266,349)	27.54 (18.22–39.95)	−2.18 (−2.32 to −2.04)
15–19 years	18,415 (10,562–27,687)	3.55 (2.03–5.33)	24,638 (14,300–36,510)	3.95 (2.29–5.85)	0.33 (0.28–0.39)
20–24 years	14,816 (8,418–22,748)	3.01 (1.71–4.62)	19,927 (11,545–30,262)	3.34 (1.93–5.07)	0.27 (0.21–0.32)
25–29 years	11,981 (7,059–18,029)	2.71 (1.59–4.07)	17,581 (10,170–26,912)	2.99 (1.73–4.57)	0.27 (0.21–0.32)
30–34 years	9,555 (5,741–14,387)	2.48 (1.49–3.73)	15,992 (9,461–23,819)	2.65 (1.57–3.94)	0.26 (0.21–0.3)
35–39 years	8,472 (5,205–12,762)	2.41 (1.48–3.62)	14,036 (8,508–21,407)	2.5 (1.52–3.82)	0.21 (0.18–0.25)
40–44 years	6,818 (4,061–10,556)	2.38 (1.42–3.68)	12,301 (7,375–19,211)	2.46 (1.47–3.84)	0.12 (0.07–0.16)
45–49 years	5,874 (3,580–9,075)	2.53 (1.54–3.91)	11,788 (6,906–18,435)	2.49 (1.46–3.89)	0.06 (−0.01 to 0.13)
50–54 years	5,019 (3,068–7,753)	2.36 (1.44–3.65)	10,903 (6,555–16,773)	2.45 (1.47–3.77)	0.15 (0.1–0.2)
55–59 years	4,261 (2,655–6,397)	2.3 (1.43–3.45)	9,426 (5,775–14,102)	2.38 (1.46–3.56)	0.15 (0.08–0.22)
60–64 years	3,310 (2,067–5,000)	2.06 (1.29–3.11)	7,271 (4,390–11,099)	2.27 (1.37–3.47)	0.23 (0.14–0.33)
65–69 years	2,332 (1,351–3,573)	1.89 (1.09–2.89)	5,764 (3,219–9,014)	2.09 (1.17–3.27)	0.46 (0.33–0.58)
70–74 years	1,510 (886–2,254)	1.78 (1.05–2.66)	3,884 (2,170–5,903)	1.89 (1.05–2.87)	0.55 (0.44–0.66)
75–79 years	853 (531–1,267)	1.39 (0.86–2.06)	2,259 (1,359–3,385)	1.71 (1.03–2.57)	0.66 (0.58–0.73)
80–84 years	405 (252–603)	1.15 (0.71–1.7)	1,174 (724–1,812)	1.34 (0.83–2.07)	0.62 (0.51–0.73)
85–89 years	136 (83–206)	0.9 (0.55–1.37)	465 (281–710)	1.02 (0.61–1.55)	0.33 (0.2–0.46)
90–94 years	29 (16–45)	0.67 (0.38–1.05)	120 (67–193)	0.67 (0.38–1.08)	−0.03 (−0.22 to 0.16)
95 + years	5 (2–9)	0.5 (0.25–0.85)	24 (11–42)	0.44 (0.21–0.78)	−0.16 (−0.36 to 0.04)
SDI region					
Low SDI	636,329 (433,125–916,268)	78.9 (53.42–113.22)	563,501 (366,834–798,764)	36.31 (23.71–51.36)	−2.66 (−2.85 to −2.47)
Low-middle SDI	856,153 (568,950–1,228,087)	52.67 (35.03–75.39)	355,962 (228,076–513,914)	18.09 (11.61–26.15)	−3.52 (−3.68 to −3.36)
Middle SDI	393,770 (258,654–555,974)	20 (13.18–28.22)	156,311 (101,981–224,243)	7.4 (4.83–10.66)	−3.24 (−3.32 to −3.17)
High-middle SDI	74,340 (49,352–106,572)	7.78 (5.17–11.16)	26,223 (16,923–37,814)	2.72 (1.69–3.95)	−3.62 (−3.75 to −3.5)
High SDI	8,565 (5,445–12,908)	1.32 (0.84–1.99)	2,247 (1,421–3,295)	0.3 (0.19–0.46)	−4.65 (−5 to −4.3)

### 3.2 Sex-specific burden

In 2021, Table 1 presents the number of the ASDR of NDs attributable to VAD for males was higher than that for females (Supplementary Figure S1). The DALYs and YLDs of NDs attributable to VAD stratified by sex were depicted in Supplementary Figure S2. The ASDR of VAD-attributable NDs for females decreased from 27.55 (95% UI: 18.35–38.74) in 1990 to 14.8 (95% UI: 9.59–21.42) in 2021, with an EAPC of −2.11 (95% *CI*: −2.29 to −1.92). And the ASDR of VAD-attributable NDs for males decreased from 37.29 (95% UI: 24.94–54.38) in 1990 to 16.6 (95% UI: 10.58–23.33) in 2021, with an EAPC of −2.68 (95% *CI*: −2.88 to −2.48) (Supplementary Figure S3). Trends in the DALYs and YLDs of VAD-attributable NDs stratified by sex were depicted in Supplementary Figure S4.

### 3.3 Age-specific burden

Table 2 provides the DALYs and ASDR of NDs attributable to VAD demonstrated significant variation across age strata. In 2021, the ASDR of NDs attributable to VAD decreased with age strata, peaking significantly in children under 5 years old [72.72 (95% UI: 46.85–105.1)] (Supplementary Figure S5). The DALYs and YLDs of VAD-attributable NDs stratified by age were depicted in Supplementary Figure S6. Longitudinal analysis (1990–2021) revealed the most rapid increase in the ASDR of VAD-attributable NDs occurred in the 75–79 age group (EAPC = 0.66, 95% *CI*: 0.58–0.73), while the steepest decline of the ASDR occurred in the <5 age group (EAPC = -2.99, 95% *CI*: −3.25 to −2.72) (Supplementary Figure S7). Trends in the DALYs and YLDs of VAD-attributable NDs stratified by age were depicted in Supplementary Figure S8.

### 3.4 Regional and national burden

As shown in Table 1, the ASDR of VAD-attributable NDs was negatively correlated with the SDI of a region. In 2021, the highest ASDR of occurred in the low-SDI region [36.31 (95% UI: 23.71–51.36)] (Supplementary Figure S9). The results presented in Table 2 demonstrate the DALYs and YLDs of VAD-attributable NDs stratified by SDI region were depicted in Supplementary Figure S10. From 1990 to 2021, the ASDR showed a declining trend across all SDI regions. The fastest decline of the ASDR occurred in the high-SDI region, with an EAPC of −4.65 (95% *CI*: −5 to −4.3) (Supplementary Figure S11). Trends in the DALYs and YLDs of VAD-attributable NDs stratified by SDI region were depicted in Supplementary Figure S12.

**Figure 2:**
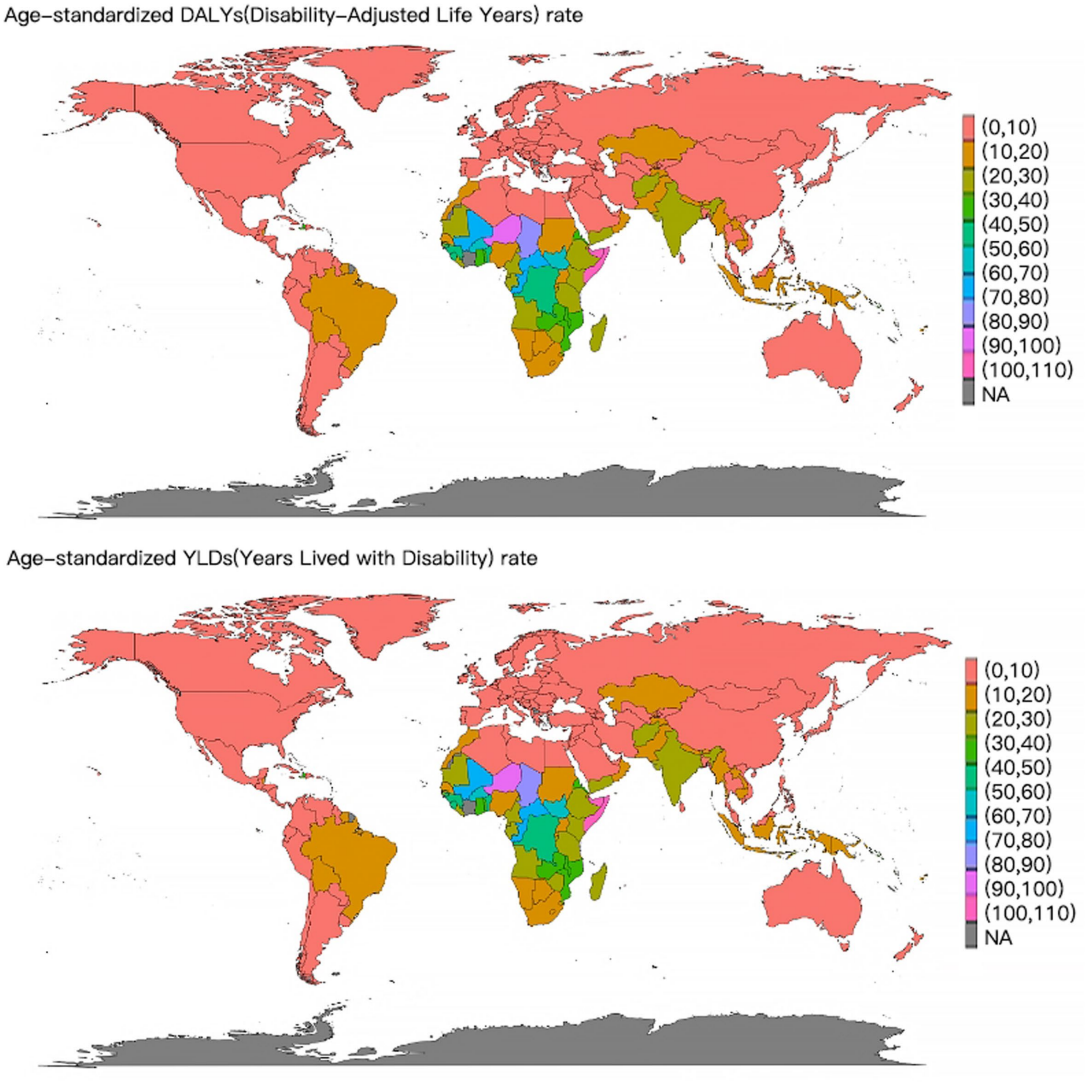
World map of the ASDR and ASYR of nutritional deficiencies attributable to vitamin A deficiency in 2021.

**Figure 3:**
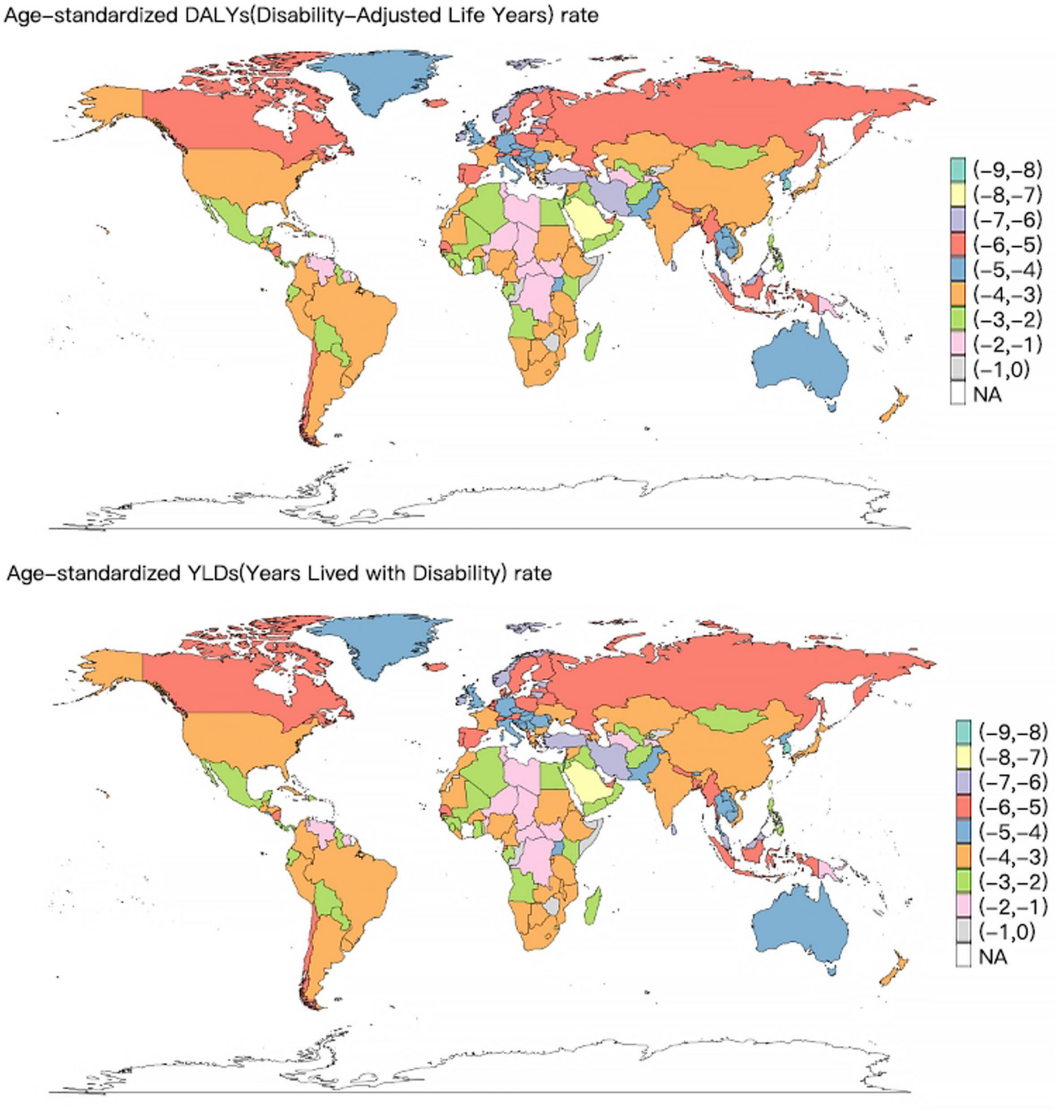
World map of the EAPC of the ASDR and ASYR of nutritional deficiencies attributable to vitamin A deficiency from 1990 to 2021.

The top three countries with the highest ASDR for VAD-attributable NDs in 2021 were Somalia [108.6 (95% UI: 68.66–169.75)], Niger [94.12 (95% UI: 61.09–136.29)] and Chad [74.27 (95% UI: 46.6–109.78)] (Figure 2). The world map of the DALYs and YLDs of NDs attributable to VAD was depicted in Supplementary Figure S13. Globally, from 1990 to 2021, the ASDR of VAD-attributable NDs decreased across all 204 countries and regions. And the fastest decline of the ASDR occurred in Maldives (EAPC = -9.8, 95% *CI*: −10.38 to −9.22), Republic of Korea (EAPC = -9.51, 95% *CI*: −10.3 to −8.72) and Taiwan (Province of China) (EAPC = -9.12, 95% *CI*: −9.87 to −8.35) (Figure 3).

### 3.5 Projections to 2050

**Figure 4:**
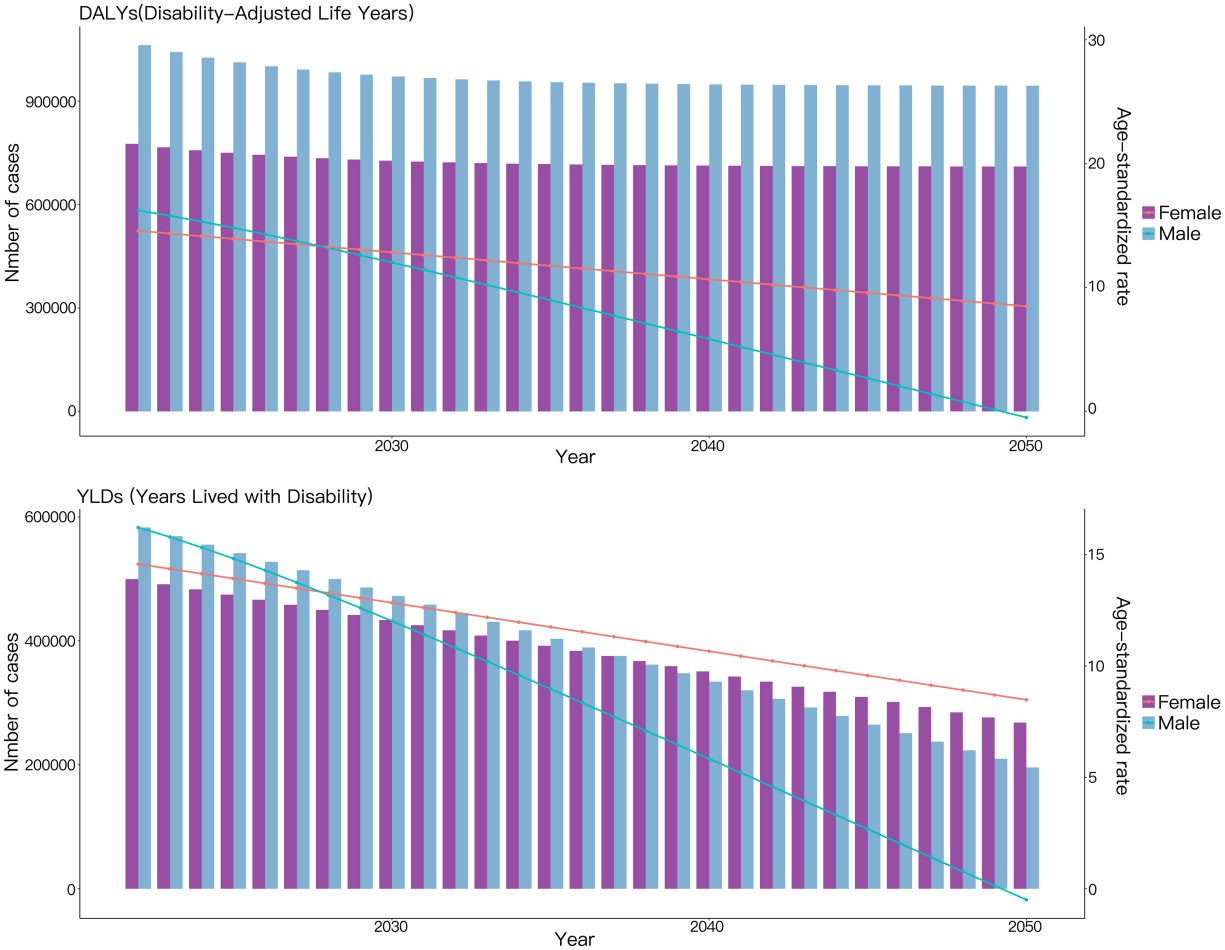
Projections to 2050 of the global burden of nutritional deficiencies attributable to vitamin A deficiency performed using the ARIMA Model.

Based on the ARIMA model predictions, from 2022 to 2050, the DALYs caused by VAD-attributable NDs are projected to show a slight decrease for both males and females, though the downward trend will be steeper among males. Additionally, the ASDR due to VAD-attributable NDs is expected to decline linearly for both genders during this period, with males demonstrating a more rapid rate of reduction compared to females (Figure 4).

**Figure 5:**
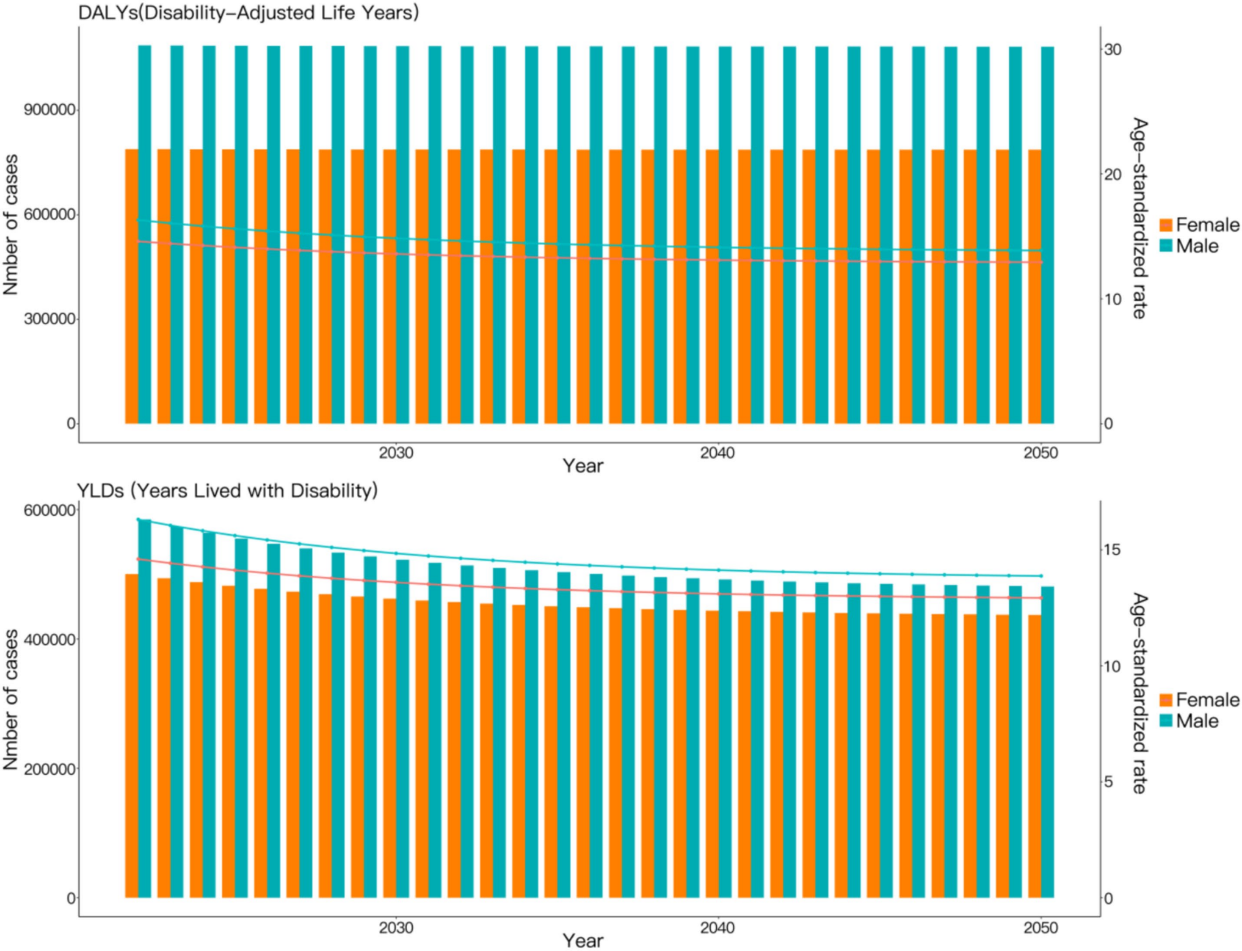
Projections to 2050 of the global burden of nutritional deficiencies attributable to vitamin A deficiency performed using the ES Model.

According to the prediction results of the ES model, differing from the predictions of the ARIMA model, the DALYs caused by NDs attributable to VAD are projected to remain almost unchanged for both males and females between 2022 and 2050. Additionally, the ASDR due to NDs attributable to VAD is expected to show only a slight decline during this period (Figure 5).

## 4 Discussion

NDs remain a pressing worldwide health concern and pose a significant global public health burden, characterized by persistent disparities across geographical regions, age demographics, and socioeconomic strata (31, 32). Building on the evidence from the 2019 GBD study on VAD, this analysis extends to broader NDs trends using updated 2021 GBD data, emphasizing the evolving dynamics of undernutrition, micronutrient deficiencies, and their intersection with emerging issues such as diet-related non-communicable diseases (NCDs) (33, 34). While significant progress has been made in reducing specific forms of malnutrition such as VAD, 2021 data highlight the complex etiology of malnutrition, particularly in many LMICs (35, 36). This discussion synthesizes the latest evidence, identifies persistent gaps, and proposes integrated strategies to address NDs in all its forms.

Vitamin A serves as a crucial nutrient, necessary for preserving visual acuity, strengthening immune defenses, maintaining dermal health, and ensuring proper cellular development (16, 37, 38). Dietary vitamin A can be obtained through both animal sources (like liver and dairy products) and plant sources (like fruits and vegetables), which contain compounds of provitamin A carotenoids (39). Vitamin A deficiency manifests through a spectrum of health issues, including dry skin, night blindness, increased susceptibility to infections, and, in severe cases, permanent blindness and death (40). The WHO recommends daily intakes for vitamin A are 900 mcg for adult men and 700 mcg for adult women to prevent deficiency. In many low-income countries, vitamin A deficiency remains widespread, primarily due to inadequate dietary intake, limited access to fortified foods, and lack of healthcare infrastructure (4, 41, 42). In contrast, overconsumption of vitamin A, often through supplementation, is more common in high-income countries and can lead to toxicity and adverse health effects, such as liver damage and bone fragility (43, 44). Addressing VAD is a critical component of global health strategies, with many nations incorporating vitamin A supplementation programs into national health policies to reduce the burden of related diseases (45).

Children under five remained the most vulnerable group to NDs-related morbidity and mortality (46). In 2021, over 45% of VAD DALYs globally were attributable to this age group, with the highest burden concentrated in sub-Saharan Africa. Early-life deficiencies have lifelong consequences, impairing cognitive development, immune function, and economic productivity (47, 48). The 2021 data highlight a troubling stagnation in reducing stunting and wasting in low-SDI regions, suggesting that current interventions—while effective—are insufficient to address the scale of need. Gender disparities further complicated malnutrition outcomes. Males consistently exhibit higher VAD DALY rates. This may be related to the fact that men generally have a higher basal metabolic rate (BMR), leading to an increased demand for fat-soluble vitamins such as vitamin A, while actual dietary intake often falls short of this requirement. Additionally, occupational exposures (e.g., outdoor labor) and unhealthy lifestyle habits (e.g., smoking, excessive alcohol consumption) may accelerate the depletion of vitamin A reserves (10, 49). Conversely, females face unique risks, including cultural practices favoring male children in resource-limited settings, and sex-specific interactions between supplementation programs and infectious diseases, which affects 30% of women globally (49–52). Adolescence emerges as a critical window for intervention, as nutritional deficits during this period perpetuate intergenerational cycles of malnutrition (53).

Globally, age-standardized rates of VAD incidence and DALYs continued their downward trajectory from 1990 to 2021, reflecting sustained efforts in food fortification, supplementation programs, and improved healthcare access. However, this overall decline conceals significant regional disparities, with low- and middle-income regions-particularly Sub-Saharan Africa and South Asia-experiencing disproportionately higher rates of VAD, still account for over 80% of the global VAD burden (10, 78). The 2021 data reveal that socioeconomic development, as measured by SDI, remains the strongest predictor of NDs outcomes (55). Countries with low SDI values, such as Somalia, Niger, and Mali, exhibit age-standardized VAD incidence rates 30–50 times higher than high-SDI nations like Australia and France (10). These disparities are exacerbated by overlapping crises, including climate change, political instability, and the lingering socioeconomic impacts of the COVID-19 pandemic, which disrupted supply chains and healthcare services in vulnerable regions (54, 56, 57). Although no abrupt changes or trend shifts in the burden of VAD were observed during the COVID-19 pandemic period (2020–2021), and direct evidence remains insufficient to confirm the pandemic’s impact on VAD burden, a limited single-center experience conducted in 2023 since the pandemic has reported significantly lower vitamin A levels in COVID-19 patients (58). This suggests that the influence of the COVID-19 pandemic on VAD burden may exhibit a delayed effect. While undernutrition rates have declined marginally, micronutrient deficiencies (e.g., iron, zinc, and iodine) persist at alarming levels, affecting an estimated 2 billion people worldwide (59). Concurrently, obesity and diet-related NCDs are rising rapidly, even in regions grappling with undernutrition (60). This dual burden reflects systemic failures in food systems, where calorie-dense, nutrient-poor diets dominate due to urbanization, market shifts toward processed foods, and inadequate policies to promote nutrient-rich agricultural production (61, 62).

Poverty, inadequate education, and limited healthcare access are root causes of NDs (63). In low-SDI regions, over 60% of households cannot afford diverse diets rich in vitamins and minerals (64). Food insecurity is compounded by reliance on staple crops with low micronutrient bioavailability, such as maize and rice (65). Even in regions where supplementation programs (e.g., vitamin A capsules) are implemented, coverage remains inconsistent due to logistical challenges, cultural resistance, and funding shortfalls (66). Economic downturns pushed an additional 100 million people into extreme poverty, reducing access to nutritious foods (67). Post-pandemic recovery efforts have largely prioritized economic stabilization over nutrition, risking further divergence between high- and low-SDI regions (68). Beyond economic and educational factors, local dietary cultures profoundly shape nutritional status. Dietary patterns are inherently shaped by the interplay of traditions, customs, and food accessibility. Alongside economic constraints, it can similarly limit access to animal-derived foods containing preformed vitamin A and plant-based foods rich in provitamin A carotenoids (69).

Approximately two-thirds of the 10.8 million child deaths that presently occur can be prevented by available interventions of which vitamin A supplementation (VAS) is one (70–72). Early reports indicated that improved vitamin A status was projected to prevent approximately 1–2 million deaths annually among children aged 1–4 years (73). Randomized controlled trials (RCTs) and cluster-RCTs also supported that VAS was associated with a clinically meaningful reduction in morbidity and mortality in children (8). However, these findings remain subject to certain challenges. Differing from the GBD 2019, VAS was omitted as a covariate in the VAD model due to its lack of statistical significance in the Spatio-Temporal Gaussian Process Regression (ST-GPR) model in GBD 2021and an implausible temporal trend was also observed when the coverage of VAS was included as a covariate in the VAD ST-GPR model (74). This highlights the highly complex nature of evaluating the real-world effectiveness of public health interventions. Although RCTs have established the biological efficacy of VAS, assessing its impact at the population level can be confounded by factors such as data quality and socioeconomic variables. Addressing inequities is equally critical, particularly among marginalized populations, where gender-responsive programs empowering women can drive improved household nutrition (75, 76). Additionally, leveraging disaggregated data and real-time monitoring systems can enhance targeted interventions, while machine learning models predicting NDs risk based on environmental and socioeconomic variables remain underutilized despite their potential (77).

While the GBD 2021 data provide valuable insights into the global burden of NDs attributable to VAD, several limitations warrant consideration. First, underreporting and diagnostic variability in LMICs may lead to an underestimate of VAD prevalence, particularly in conflict zones and remote areas (41). Second, the SDI framework, while useful for identifying high-burden regions, oversimplifies the multidimensional drivers of NDs, such as cultural factors and intra-country disparities (20). Future studies should incorporate granular indicators, such as household dietary diversity and women’s empowerment metrics, to refine inequality analyses. Third, this study thoroughly examines the global and stratified disease burden of NDs attributed to VAD. However, when applying the data to specific regions, caution is needed. Further research is needed to clarify the regional burden of VAD-attributable NDs. In addition, a limitation of our analysis is the absence of formal goodness-of-fit statistics (e.g., AIC/BIC) and cross-validation. This choice was motivated by our goal to present complementary forecasting scenarios rather than to select a superior model. We suggest that subsequent studies could further compare predictions from different models (e.g., ARIMA and ES) to generate more robust forecasting outcomes.

## 5 Conclusion

The GBD 2021 data highlight the complexity of addressing VAD as a global public health challenge. Despite the burden of VAD in high-income nations having been significantly reduced, LMICs remain a critical focus for global health efforts. Addressing the disparities in VAD prevalence requires comprehensive strategies combining targeted interventions, policy reforms, and cross-sectional collaboration. While the GBD offers extensive coverage, its temporal constraints and potential underreporting in conflict-affected regions and health system-weak areas highlight the need for additional data collection to enhance predictive accuracy. By leveraging the insights from the GBD 2021 data, future research and policy initiatives can better inform the development of sustainable strategies to combat VAD and improve the health outcomes of vulnerable populations worldwide.

## Data Availability

Publicly available datasets were analyzed in this study. This data can be found here: https://vizhub.healthdata.org/gbd-results/.
