# Supplementary material for: A comprehensive analysis of vitamin a deficiency burden and trends: insights from the global burden of disease study 2021 and future predictions to 2050
Source: Front Nutr. 2025 Nov 18;12:1673576. doi: 10.3389/fnut.2025.1673576 (PMC12671203; doi:10.3389/fnut.2025.1673576)
Supplement: Supplementary file 1 [file Image_1.PDF]

Supplementary Materials

Supplementary Figure 1

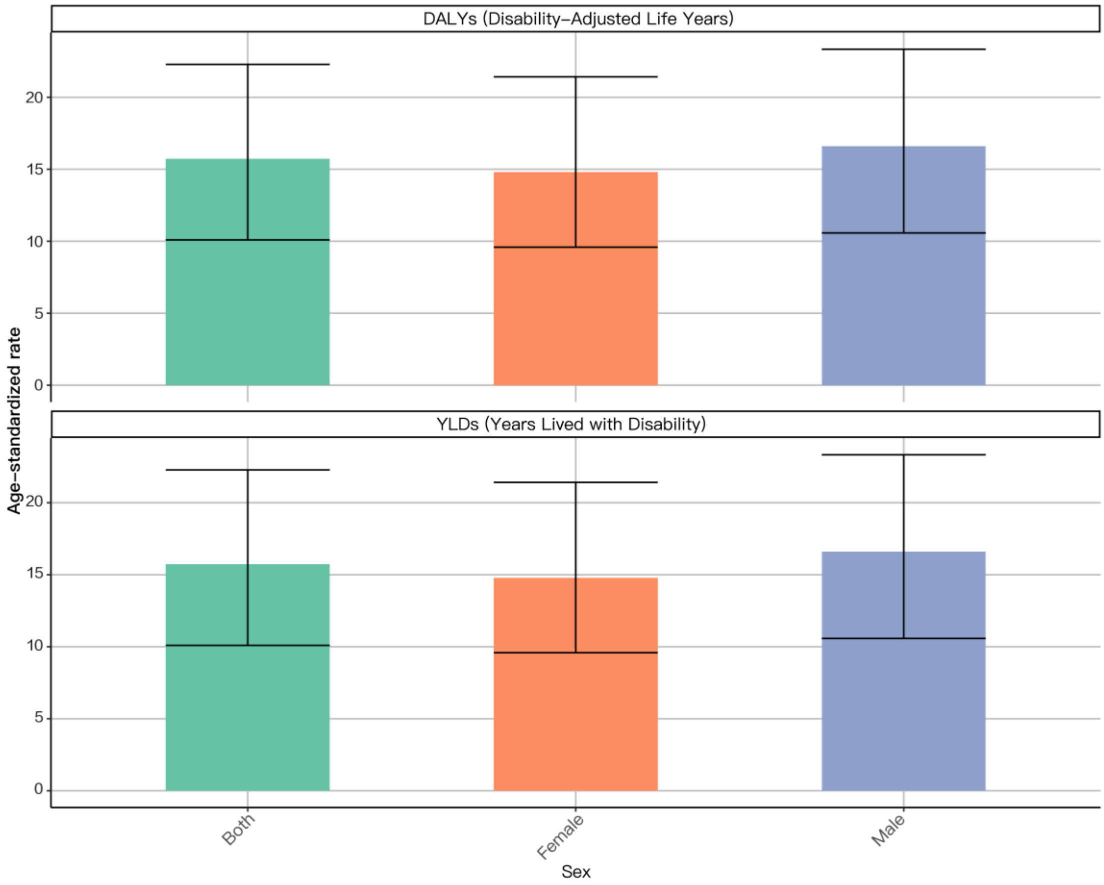

Supplementary Figure 1: The ASDR and ASYR of nutritional deficiencies attributable to vitamin A deficiency by sex in 2021.

Supplementary Figure 2

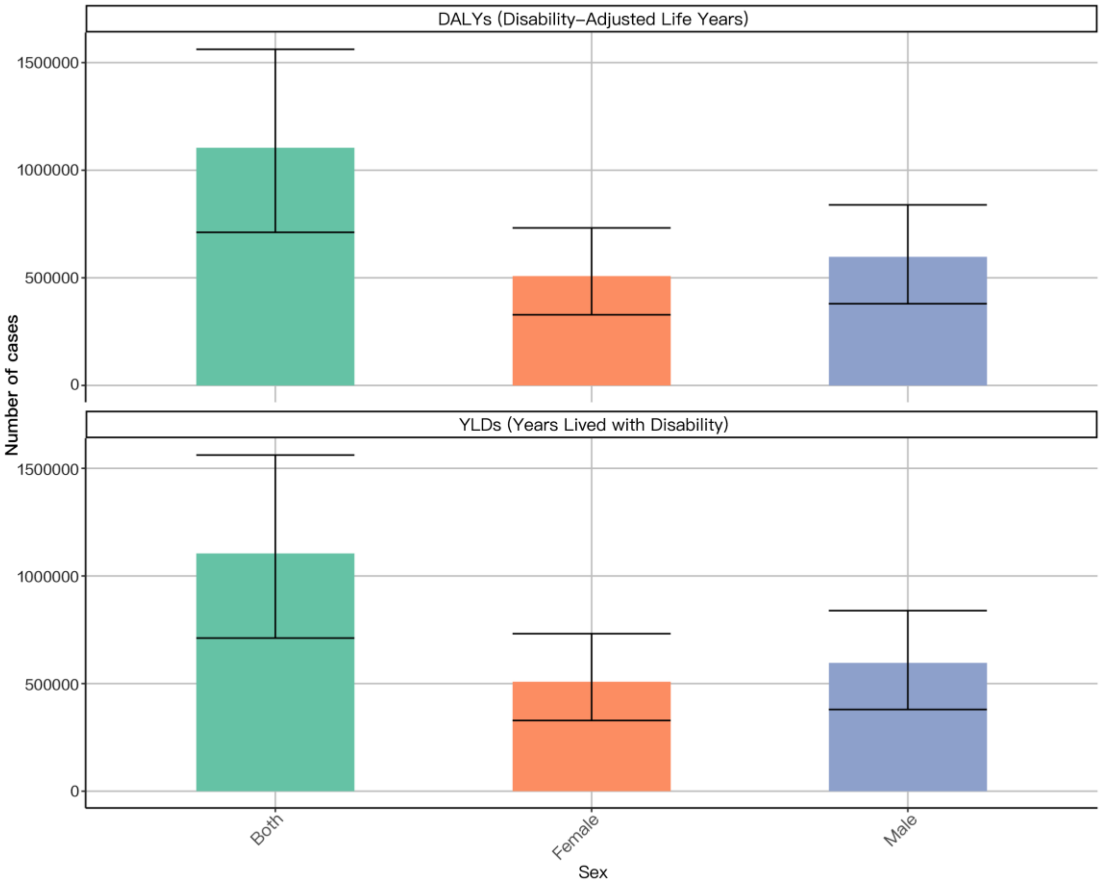

Supplementary Figure 2: The DALYs and YLDs of nutritional deficiencies attributable to vitamin A deficiency by sex in 2021.

Supplementary Figure 3

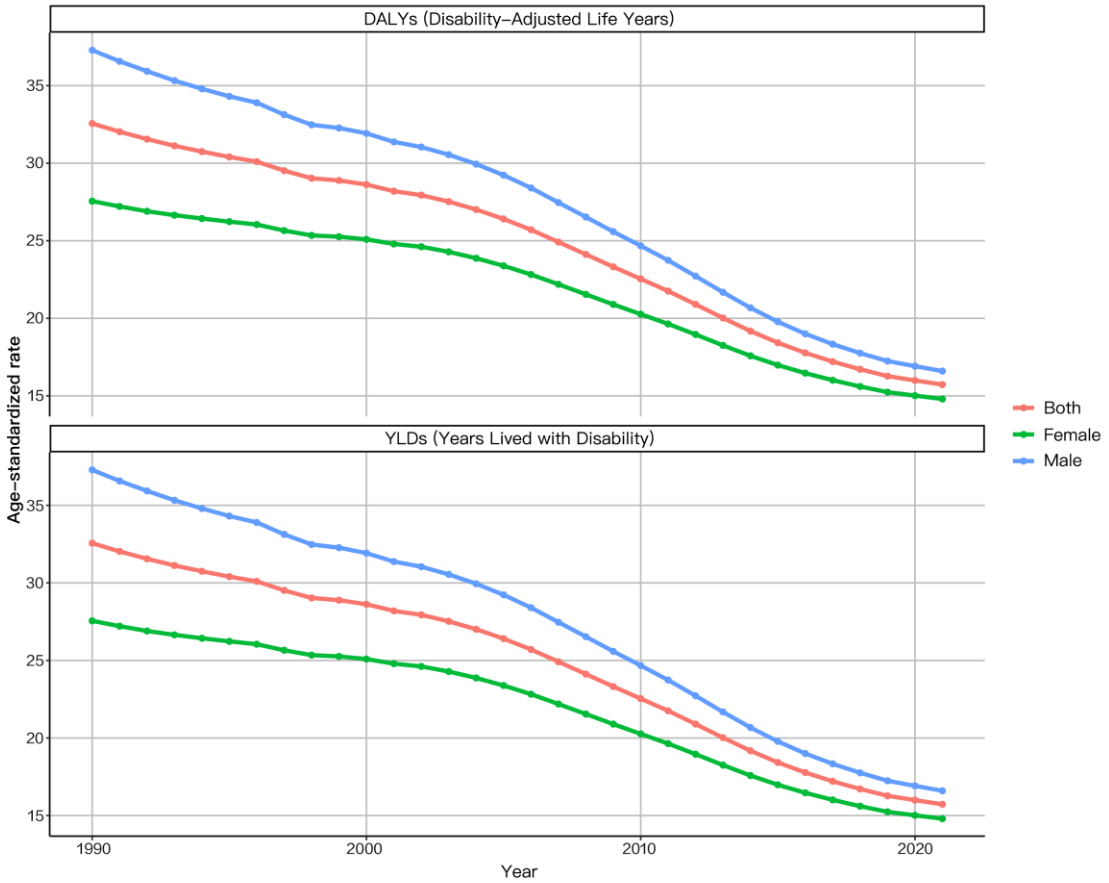

Supplementary Figure 3: Trends in the ASDR and ASYR of nutritional deficiencies attributable to vitamin A deficiency by sex from 1990 to 2021.

Supplementary Figure 4

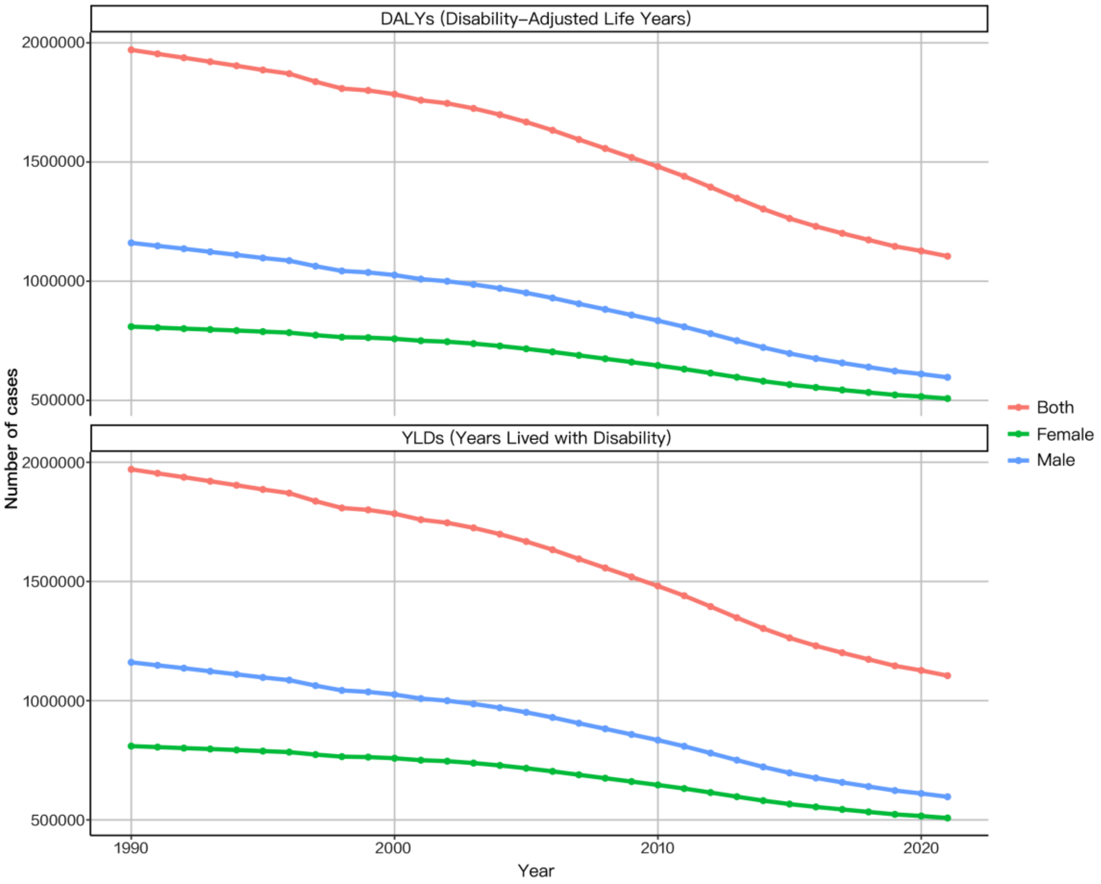

Supplementary Figure 4: Trends in the DALYs and YLDs of nutritional deficiencies attributable to vitamin A deficiency by sex from 1990 to 2021.

Supplementary Figure 5

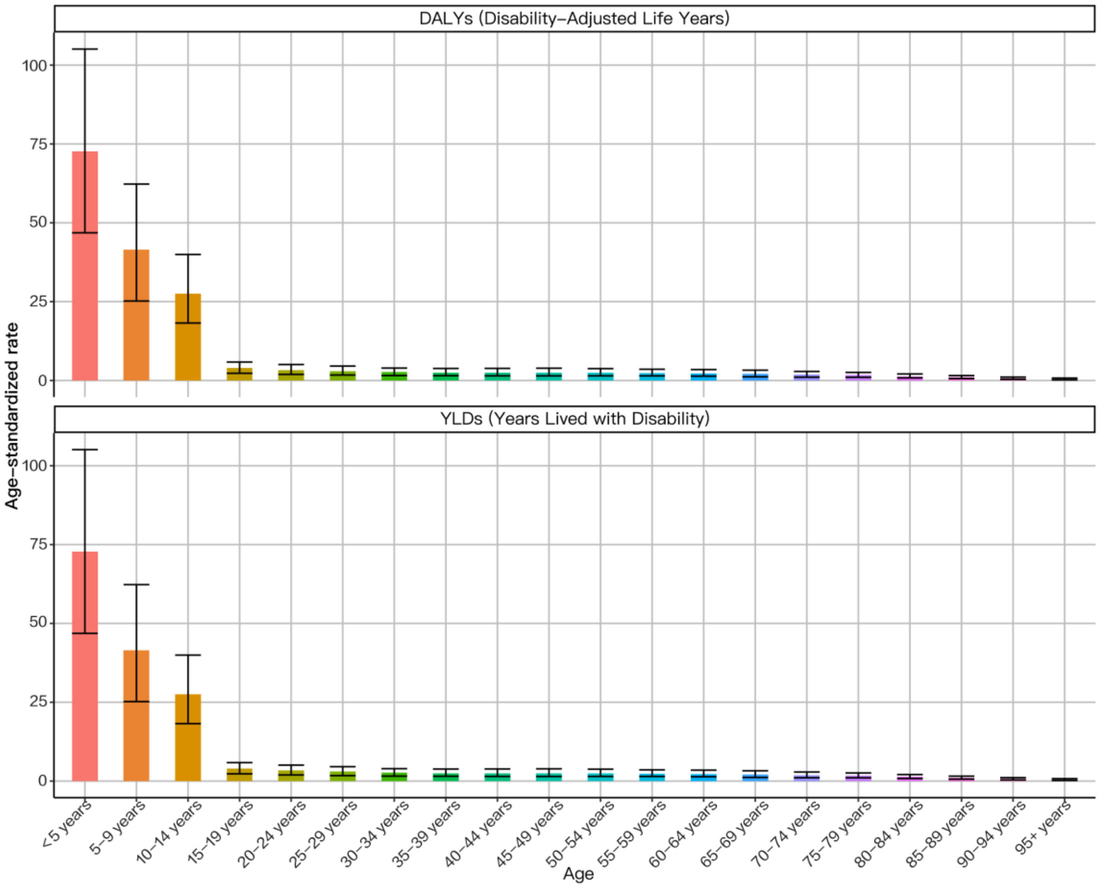

Supplementary Figure 5: The ASDR and ASYR of nutritional deficiencies attributable to vitamin A deficiency by age in 2021.

Supplementary Figure 6

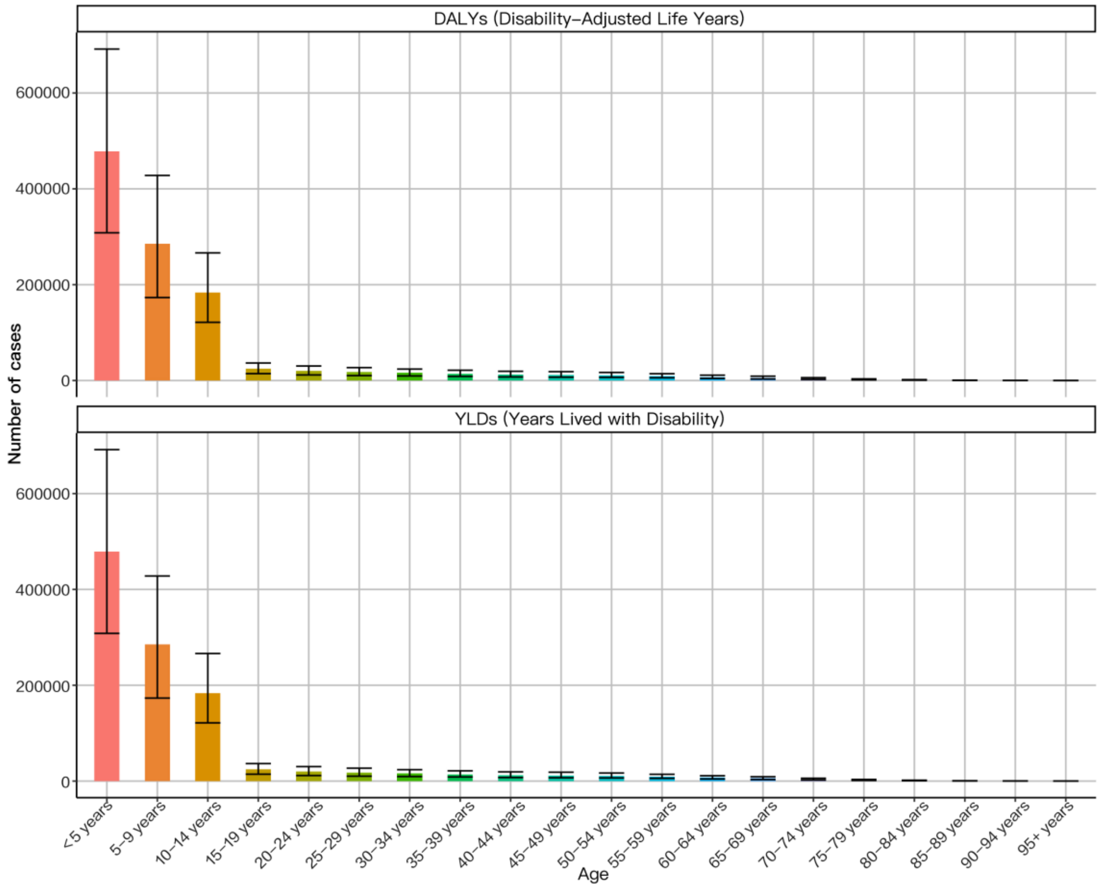

Supplementary Figure 6: The DALYs and YLDs of nutritional deficiencies attributable to vitamin A deficiency by age in 2021.

Supplementary Figure 7

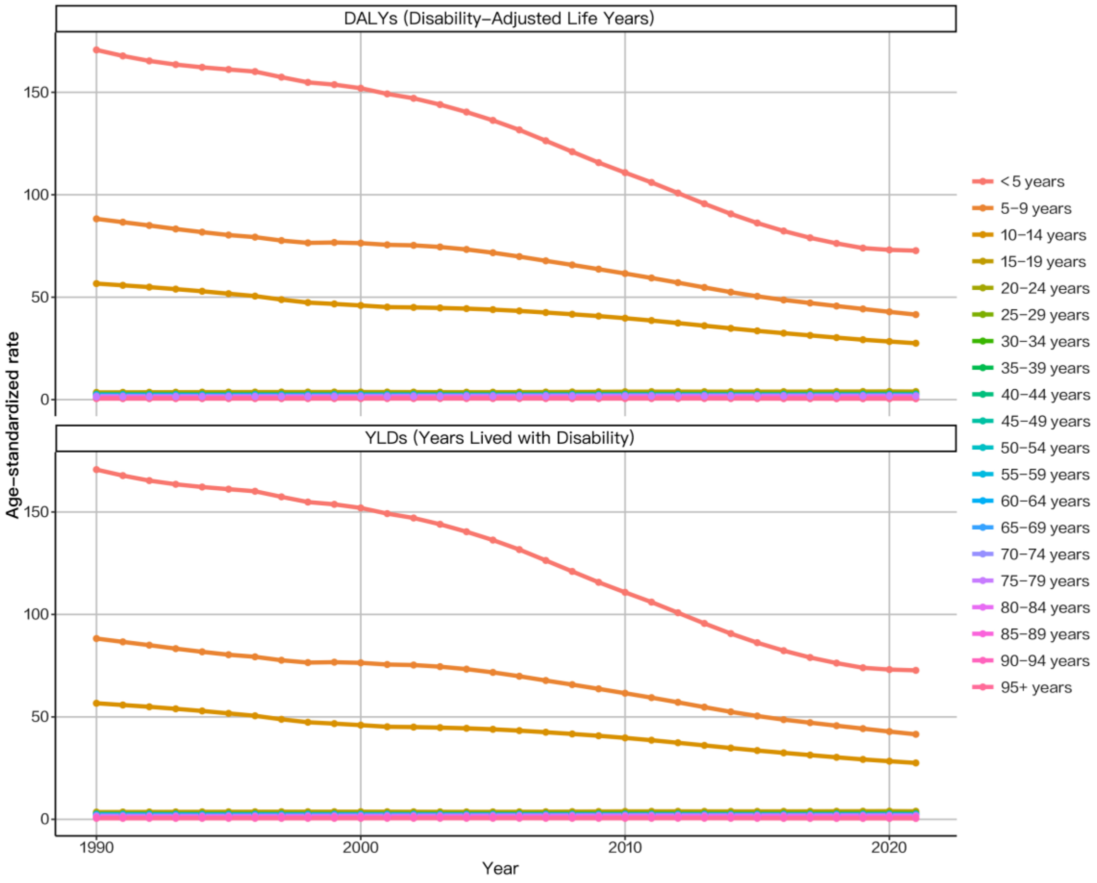

Supplementary Figure 7: Trends in the ASDR and ASYR of nutritional deficiencies attributable to vitamin A deficiency by age from 1990 to 2021.

Supplementary Figure 8

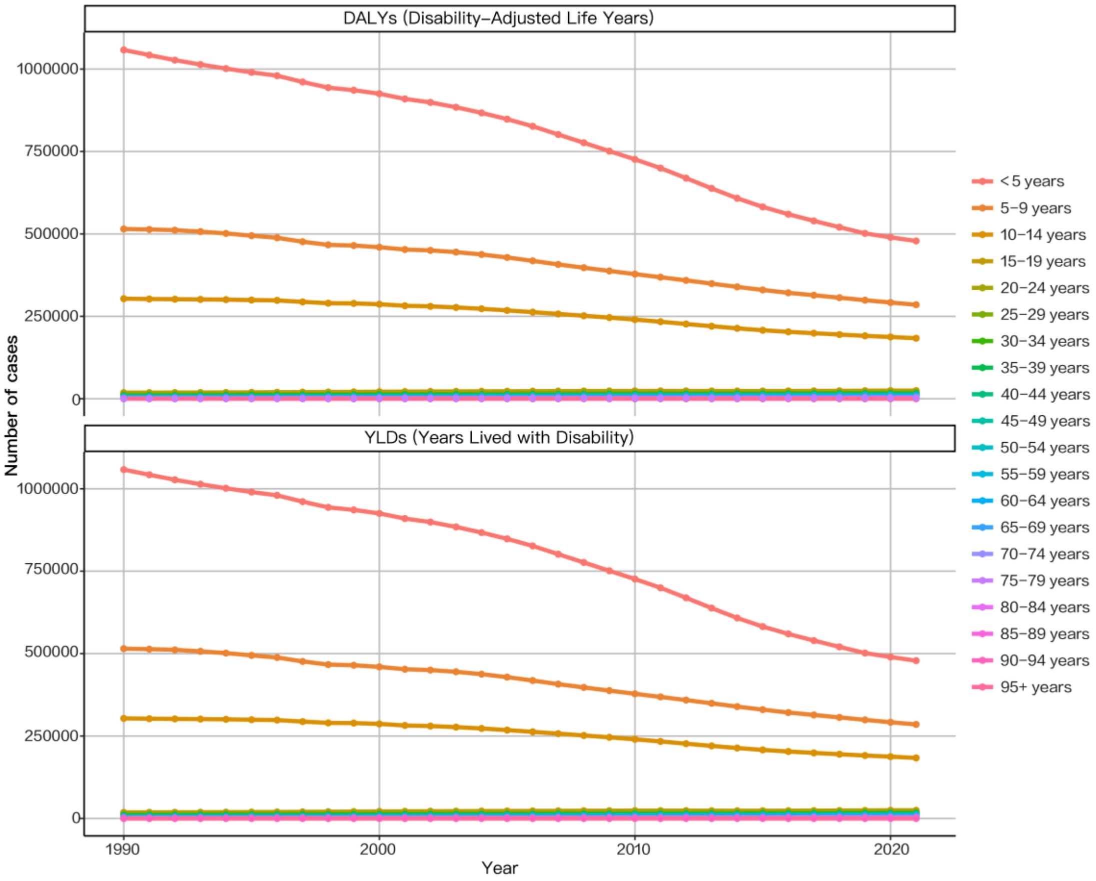

Supplementary Figure 8: Trends in the DALYs and YLDs of nutritional deficiencies attributable to vitamin A deficiency by age from 1990 to 2021.

Supplementary Figure 9

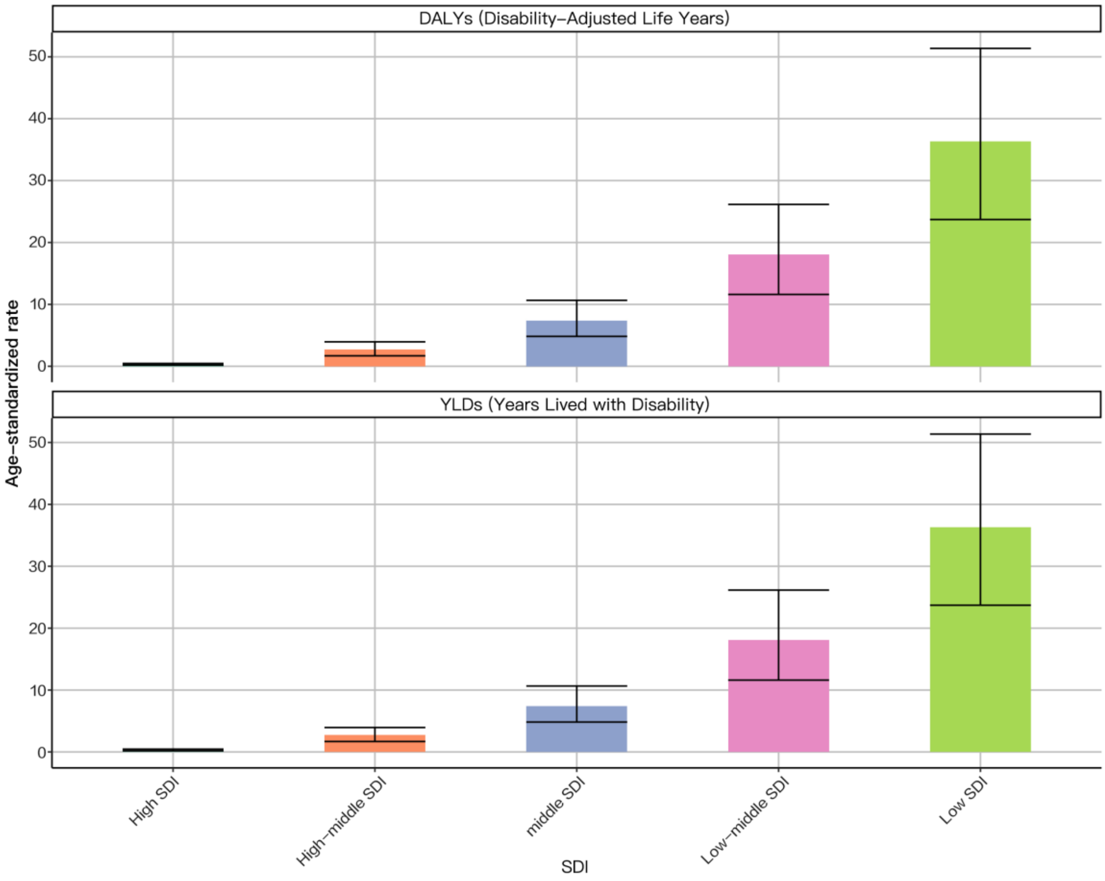

Supplementary Figure 9: The ASDR and ASYR of nutritional deficiencies attributable to vitamin A deficiency by age in 2021.

Supplementary Figure 10

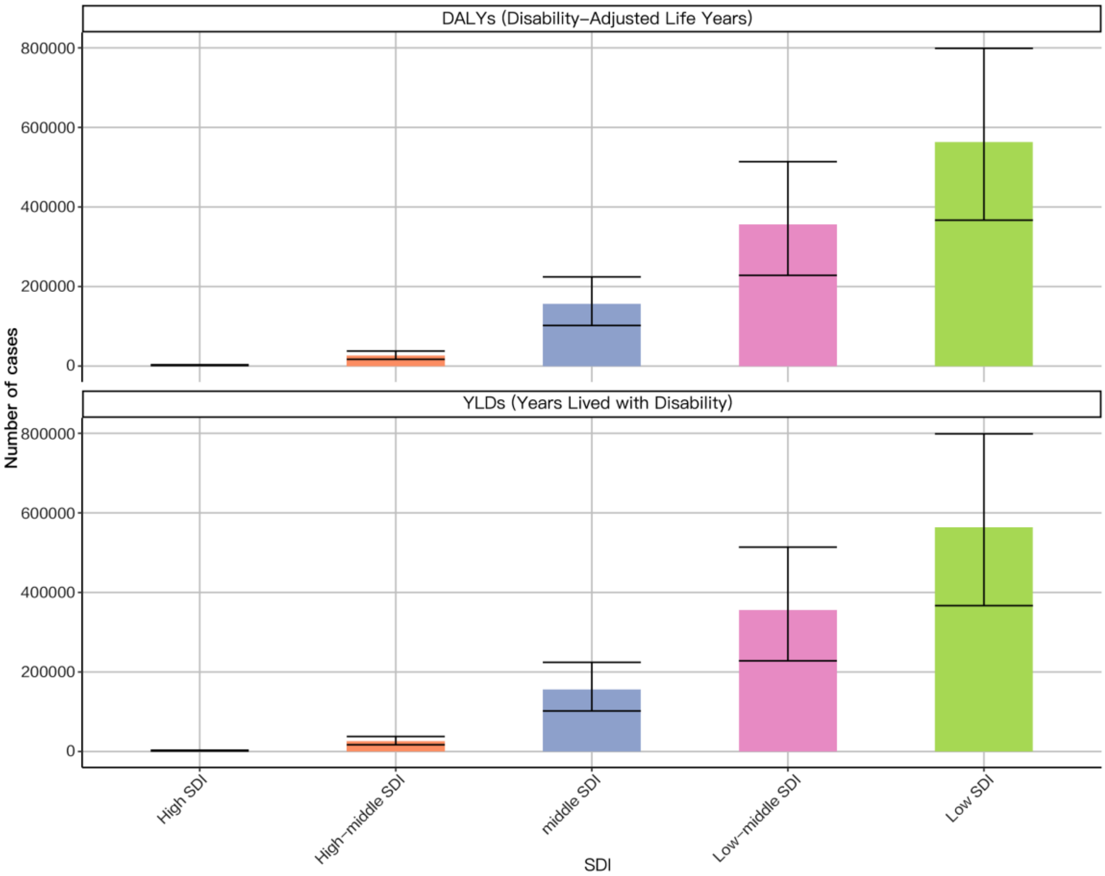

Supplementary Figure 10: The DALYs and YLDs of nutritional deficiencies attributable to vitamin A deficiency by SDI region in 2021.

Supplementary Figure 11

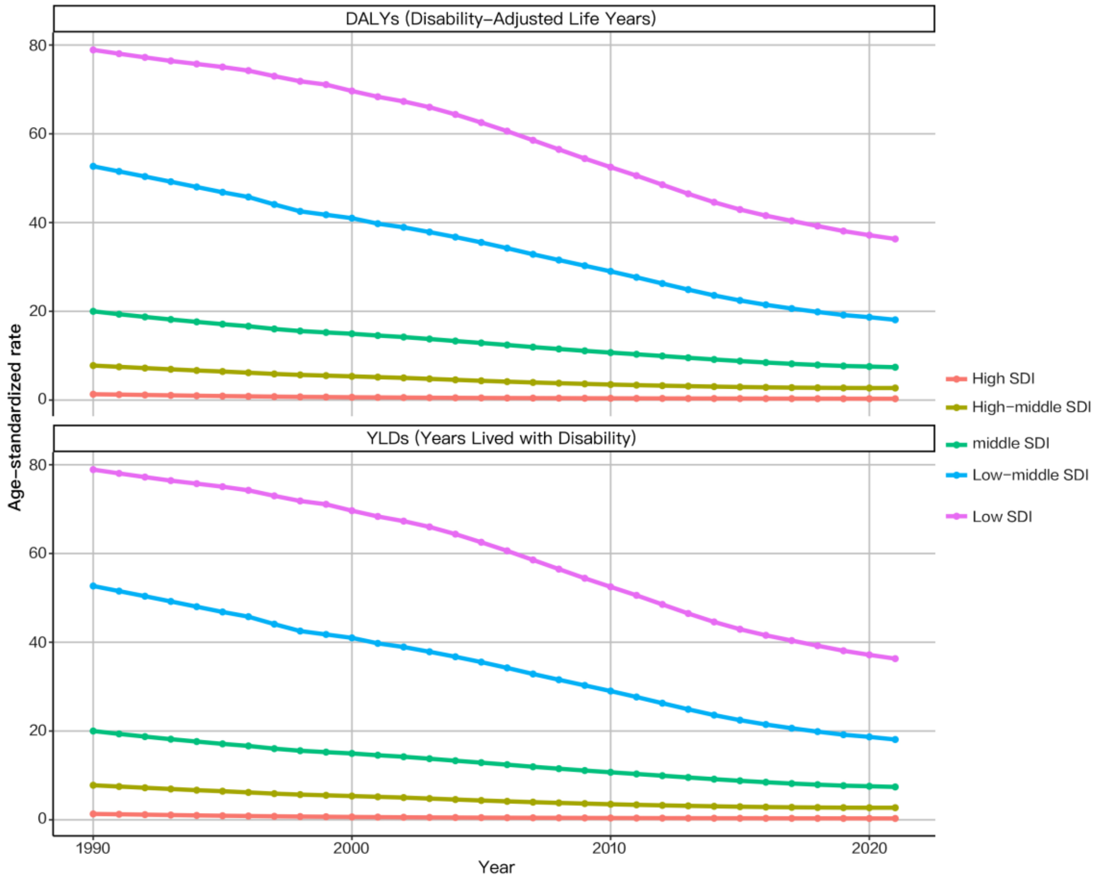

Supplementary Figure 11: Trends in the ASDR and ASYR of nutritional deficiencies attributable to vitamin A deficiency by SDI region from 1990 to 2021.

Supplementary Figure 12

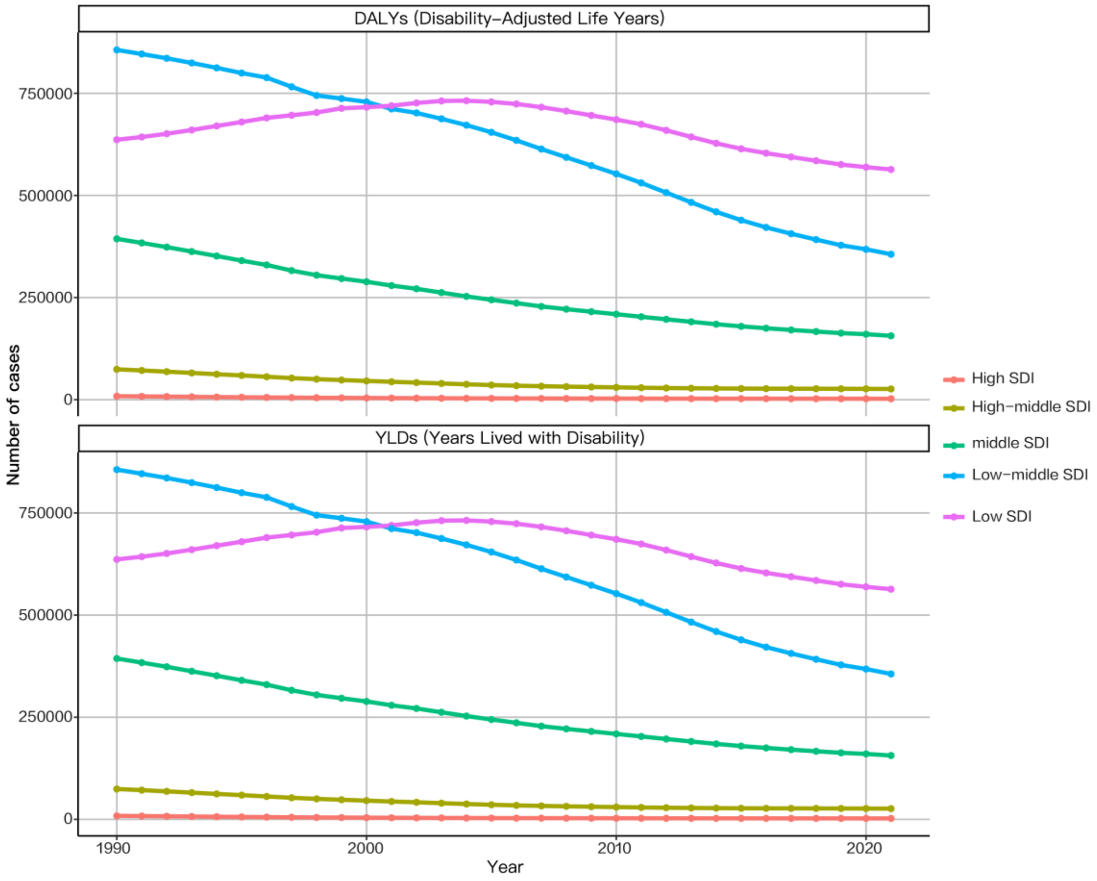

Supplementary Figure 12: Trends in the DALYs and YLDs of nutritional deficiencies attributable to vitamin A deficiency by SDI region from 1990 to 2021.

Supplementary Figure 13

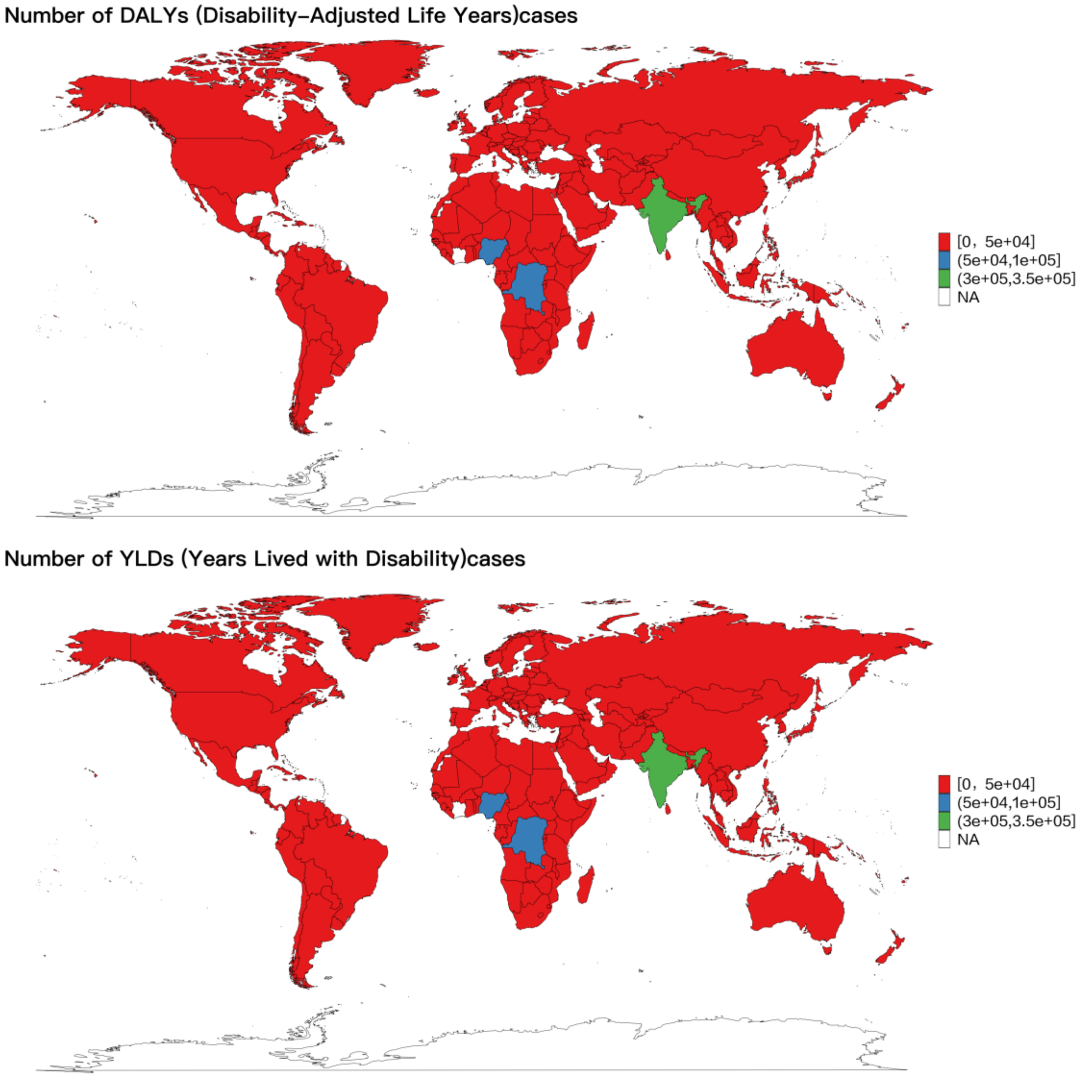

Supplementary Figure 13: World map of the DALYs and YLDs of nutritional deficiencies attributable to vitamin A deficiency in 2021.
